# Long, elliptically bent, active X-ray mirrors with slope errors <200 nrad

**DOI:** 10.1107/S1600577517005422

**Published:** 2017-04-19

**Authors:** Ioana T. Nistea, Simon G. Alcock, Paw Kristiansen, Adam Young

**Affiliations:** a Diamond Light Source, Harwell Science and Innovation Campus, Didcot, Oxfordshire OX11 0DE, UK; b FMB Oxford Ltd, Oxford OX2 0ES, UK

**Keywords:** X-ray active optics, mechanically bent mirrors, optical metrology, Diamond-NOM

## Abstract

Actively bent X-ray mirrors are important components of many synchrotron and X-ray free-electron laser beamlines. A high-quality optical surface and good bending performance are essential to ensure that the X-ray beam is accurately focused. Two elliptically bent X-ray mirror systems from FMB Oxford were characterized in the optical metrology laboratory at Diamond Light Source. A comparison of Diamond-NOM slope profilometry and finite-element analysis is presented to investigate how the 900 mm-long mirrors sag under gravity, and how this deformation can be adequately compensated using a single, spring-loaded compensator. It is shown that two independent mechanical actuators can accurately bend the trapezoidal substrates to a range of elliptical profiles. State-of-the-art residual slope errors of <200 nrad r.m.s. are achieved over the entire elliptical bending range. High levels of bending repeatability (Δ*R*/*R* = 0.085% and 0.156% r.m.s. for the two bending directions) and stability over 24 h (Δ*R*/*R* = 0.07% r.m.s.) provide reliable beamline performance.

## Abstract 2

Optical metrology and finite-element analysis studies of two actively bent X-ray mirrors are presented. Each 900 mm-long mirror can be mechanically bent to a range of elliptical profiles with state-of-the-art slope errors of <200 nrad r.m.s.

## 1. Introduction   

Grazing-incidence X-ray mirrors are used extensively at all synchrotron radiation and free-electron laser (FEL) facilities to focus or collimate intense photon beams for scientific research. Most beamlines can be configured to suit experimental requirements for a variety of applications. As such, most beamlines require an adjustable optical arrangement to match the diameter of the X-ray beam to the size of the sample, or alternatively to vary the focal distance to suit different sample or detector positions. This can be achieved either by using active optics or a transfocator containing a user-selectable number of compound refractive X-ray lenses. Active optics with deformable reflective surfaces, such as piezo bimorph deformable mirrors (Alcock *et al.*, 2015 ▸) or mechanically bent mirrors, enable focusing parameters of the beamline to be easily and quickly adjusted. The simplest form of mechanical bender employs a single actuator to apply equal forces to the ends of a cuboid-shaped substrate, thereby inducing a cylindrical bend in the optical surface. A unique elliptical profile can be created using a one-moment bender and a substrate with a carefully chosen trapezoidal width (or depth) (Padmore *et al.*, 1996 ▸). A more sophisticated two-moment system can bend a substrate to a range of ellipses by applying different forces to each end of the mirror. At modern low-emittance synchrotron radiation sources and FELs, the quality of X-ray optics is often a major limitation to beamline performance (Siewert *et al.*, 2014 ▸). This necessitates the creation of improved X-ray mirrors. Guided by advances in optical and X-ray metrology (Wang *et al.*, 2016 ▸), deterministic polishing techniques such as ion beam figuring (Idir *et al.*, 2015 ▸) and elastic emission machining (EEM) (Takei *et al.*, 2013 ▸) can now routinely fabricate long X-ray mirrors with slope errors <200 nrad root-mean-square (r.m.s.). Due to continuous improvements in the quality of optical surfaces, the new challenge for X-ray optics is minimizing additional slope errors introduced by mounting the substrate into its holder and adding cooling manifolds. For active optics there is also the added difficulty of ensuring that parasitic distortions (including bend hysteresis, roll errors and sagittal bending or twisting) are not induced by tangential bending. Such errors can be caused by many factors, including inadequate holding forces, flexible clamps or misalignment of force actuators. High-quality metrology instruments and ultra-stable environments are essential to aid optimization of clamping and bending to guarantee the best possible X-ray performance for beamline optics. *Ex situ* optimization and fault-finding investigations of such systems prior to beamline installation can save valuable X-ray commissioning time. We investigate whether mechanically bent X-ray mirrors can reliably and repeatedly be bent to specified ellipses, and retain their curvature for >24 h. Used in combination with experimental data, finite-element analysis (FEA) offers the possibility of predicting and improving the performance of active X-ray optics.

## 2. Experimental   

FMB Oxford, UK, have previously built several cylindrical mirror benders using a single, one-moment actuator (Vannoni *et al.*, 2016 ▸). They have recently extended this design to create two-moment actuator systems for elliptical bending of long X-ray mirrors. The mirror systems described in this paper will be installed on the 24-ID-C (‘C-line’) and 24-ID-E (‘E-line’) beamlines at the Advanced Photon Source (APS), USA.

### 2.1. Substrates   

Two Si(100) planar mirrors, each of length *L* = 900 mm and height *H* = 57.5 mm, were fabricated by Carl Zeiss SMT GmbH, Germany. Each substrate has a trapezoidal width *W* to assist with elliptical bending. The wider end of each substrate is positioned at the upstream end of the beamline. Without any form of optical clamping, and with each mirror’s surface normal pointing horizontally (to counteract gravitational sagging), the Zeiss D100 Fizeau interferometer measured a tangential slope error of <200 nrad r.m.s. and a radius of curvature flatter than 450 km. Parameters for the two substrates are listed in Table 1 ▸.

### 2.2. Bending a substrate with a trapezoidal width   

Assuming that the dimensions of an X-ray mirror permit it to be approximated as a thin beam, Bernoulli–Euler theory (Howells *et al.*, 2000 ▸; Padmore *et al.*, 1996 ▸) predicts how the height profile *y* at position *x* along a mirror of length *L* will elastically deform when bending couples *C*_1_ and *C*_2_ are applied at its ends,
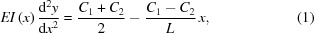
where *E* is Young’s modulus and *I*(*x*) is the moment of inertia. The second derivative of the height profile, d^2^ *y*/d*x* ^2^, the so-called ‘curvature’, is inversely proportional to the mirror’s radius of curvature *R*. If equal moments are applied (*C* = *C*_1_ = *C*_2_) to a mirror of fixed width *W* and thickness *H*, then inserting *I* = *WH* ^3^/12 into equation (1), and integrating, leads to
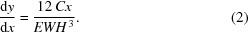
As expected, the slope profile d*y*/d*x* is proportional to *x*, proving that a mirror with a fixed rectangular cross section bends cylindrically in a one-moment bender. However, for a mirror with a trapezoidal width *W*(*x*) = *b* − *ax*, the equation for the mirror’s curvature becomes
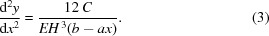
Integrating equation (3) provides the slope profile of the optical surface,
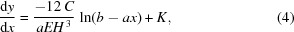
where *K* is the constant of integration. Arbitrarily defining the slope at the centre of the mirror (*x* = 0) to be zero (tilt removal), we obtain
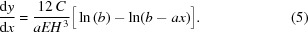
Therefore, the amount of bending (slope change) for a mirror with a trapezoidal width varies logarithmically along its length. This relationship can be utilized to find the trapezoid parameters *a* and *b* for optimally dimensioning the substrate.

### 2.3. Mechanical bender   

Each independent actuator bends the mirror about a pivot point. A centrally located spring-loaded cell provides a tunable force to counteract gravitational sag. Phytron VSS52.200.1.2 stepper motors apply independent forces to each end of the mirrors. Motors operate in closed-loop with feedback from Renishaw encoders (T2621-30M) and interpolators (Ti1000E04A). Substrates and bending mechanics were designed to provide the elliptical bending ranges shown in Table 2 ▸. FEA was performed at FMB Oxford using ANSYS R15 software to calculate the trapezoidal width profile to optimally bend each optic to a specified ellipse. Each mirror’s width was tapered such that only an additional ∼1 nrad r.m.s. is added to the tangential slope error when the mirror is bent to the nominal ellipse. A linear regression model (McKinney *et al.*, 2009 ▸) was used to calculate bending moments which minimize the slope error. Long actuator arms were purposefully chosen to provide high-resolution bending. Both mirrors are designed to operate in a vertical focusing (bounce upwards) configuration to suit the beamline geometry.

**Figure 1:**
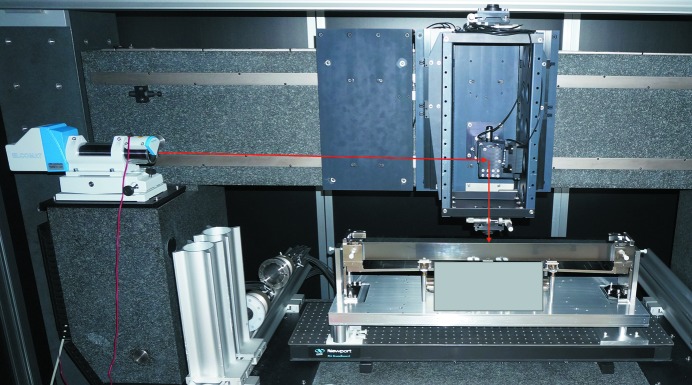
Two-moment elliptically bent mirror system from FMB Oxford, installed on the Diamond-NOM slope profiler for metrology and optimization. The bending actuators are purposefully obscured so as not to reveal the commercially sensitive design.

The two X-ray mirror systems from FMB Oxford were assembled in the Optics and Metrology cleanroom at Diamond Light Source Ltd. This environmentally stabilized laboratory contains a suite of metrology instruments capable of characterizing state-of-the-art synchrotron X-ray optics (Alcock *et al.*, 2016 ▸). After assembly, each mirror was sequentially installed, aligned and tested on the Diamond-NOM (see Fig. 1 ▸) in a face-up geometry. The Diamond-NOM (Alcock *et al.*, 2010 ▸) is a non-contact slope profiler which utilizes a high-grade pentaprism and computer-controlled air bearing stages to scan a narrow beam of light from an autocollimator (AC) in sub-millimetre steps along the length of the surface under test. Angular deflection of the light reflected from the test mirror is recorded by the AC. Height information, with sub-nanometre resolution, is extracted by integrating the slope data. A pinhole with a diameter of 3 mm is located in close proximity (<5 mm ideally) to the optic to define the size of the AC beam illuminating the optical surface. Pitch and roll of each mirror were manually adjusted to align with the AC’s beam. An environmental enclosure around the Diamond-NOM passively stabilizes air temperature fluctuations to <10 mK over several days, and also reduces excessive air flows, acoustic noise and stray light. Thermal sources, such as the controller unit for the bender motors, were purposefully located outside the enclosure to minimize the impact of heat, mechanical vibration and air current perturbances on the measurements. With such precautions, previous experiments have shown that the Diamond-NOM is capable of reliably measuring X-ray mirrors with slope errors <50 nrad r.m.s. (Alcock *et al.*, 2016 ▸). Bending motors were driven using a MCS8+ motion controller, *via* the Experimental Physics and Industrial Control System (EPICS), which enabled synchronization with Diamond-NOM scans also controlled *via* EPICS. The coordinate along the length of each mirror was defined as *x* = 0 mm at the centre of the mirror, and *x* = −450 mm at the thick upstream end. Previous studies at Diamond have shown that mirrors can take more than one day to mechanically ‘settle’ on the nanometre scale after clamping into their holders. To help speed up this relaxation process, each mirror was cycled ten times over its full bending range. Fizeau interferometry, or the variation in roll angle measured by the AC, can help to visualize, and iteratively minimize, sagittal twisting when substrates are clamped into their opto-mechanical holders.

## 3. Results   

A series of metrology tests were performed for each mirror, including: gravitational sag compensation, ellipse optimization, and bending linearity, repeatability and range. For brevity, only a single representative example of each investigation is provided below.

### 3.1. Gravitational sag and compensation   

**Figure 2:**
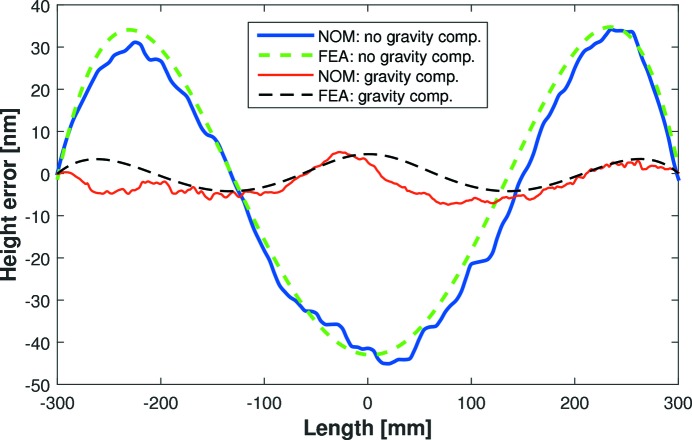
Comparison between Diamond-NOM slope profilometry (solid lines) and finite-element analysis (FEA) (dashed curves) showing how gravity affects the E-line mirror (after removal of best-fit cylinders). The larger-amplitude (blue and green) curves show the mirror’s profile before applying a central gravity compensator, as measured by the Diamond-NOM and calculated by FEA, respectively. The smaller-amplitude (red and black) curves show the mirror’s profile after applying the gravity compensator, as measured by the Diamond-NOM and calculated by FEA, respectively. Adding the gravity compensator improves the slope error from ∼500 nrad to ∼200 nrad r.m.s.

**Figure 3:**
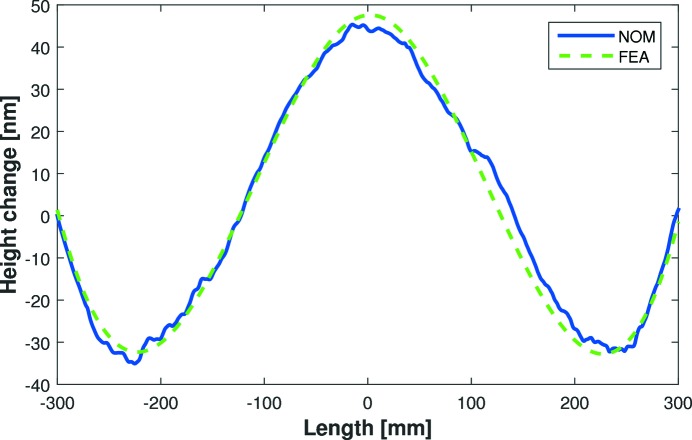
Change in the E-line mirror’s height profile (after removal of best-fit cylinder) by adding a central gravitational compensator. Diamond-NOM data (blue solid curve) are in excellent agreement with the FEA prediction (green dashed curve). Each curve is the difference between the two experimental or FEA curves shown in Fig. 2 ▸.

Euler–Bernoulli theory predicts how a thin beam supported at its ends will sag under its own weight. The height profile *y*(*x*) along its length *L* will distort according to a fourth-order polynomial relationship in *x* (Beer *et al.*, 2012 ▸),
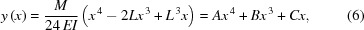
where *M* is the load per unit length. A two-moment actuator can correct third-order polynomial height errors, but cannot remove fourth-order quartics (Howells *et al.*, 2000 ▸). FEA modelling was performed by FMB Oxford to predict how each mirror sags under its own weight, with and without a centrally located gravity compensator (Ice, 1996 ▸). FEA predicts that the E-line mirror naturally sags with a fourth-order height error (after removal of a best-fit cylinder) as shown by the larger-amplitude dashed (green) curve in Fig. 2 ▸. This quartic M-shaped height error matches the prediction of equation (6). Even assuming a perfect substrate with no polishing errors, this corresponds to a slope error of ∼450 nrad r.m.s. over the central 600 mm, which is considerably larger than the beamline requirement of <200 nrad r.m.s.. FEA simulations predict that a force of 25 N applied upwards at the centre of the E-line mirror will minimize gravity-induced distortion. The improved height error after gravity compensation, as shown by the smaller amplitude dashed curve (black) in Fig. 2 ▸, corresponds to a slope error of ∼155 nrad r.m.s. To investigate these FEA predictions, the Diamond-NOM measured the mirror’s height error with and without a central compensator applying 25 N to the mirror. Fig. 2 ▸ shows that the experimental data (solid curves) are in excellent agreement with FEA (dashed curves). Even including the mirror’s polishing errors, the Diamond-NOM data confirm that the gravity compensator significantly improves the optic’s slope error from ∼500 nrad to ∼200 nrad r.m.s. Small ripples in the Diamond-NOM data in Fig. 2 ▸ are due to polishing errors on the optical surface. However, since these short-wavelength errors are intrinsic to the substrate, and are not strongly influenced by the compensator, the difference between Diamond-NOM scans of the mirror with and without gravity compensation reveals the influence of the compensator. As shown in Fig. 3 ▸, the experimental measurement of the influence of the gravity compensator (solid curve) is in excellent agreement with the fourth-order polynomial height change predicted by equation (6) and the FEA dashed curve. This confirms the benefits of using FEA predictions to guide the metrology search for an optimized X-ray mirror system.

### 3.2. Bending range and linearity   

To determine the mirror’s range of bending with the gravity compensator installed, equal counts were applied to both motors over their full working range. For the E-line mirror, the best-fit concave cylindrical radii of curvature at the positive limit, home position and negative limit were 12.4 km, 4.2 km and 2.1 km, respectively. Similarly, the C-line mirror could be bent from 11.6 km to 1.7 km. Both mirror’s motors have a full range of ∼3300k encoder counts, of which it is predicted that only the central ∼1000k counts are necessary to achieve the specified range of ellipses, corresponding to radii of curvature of 3 to 4 km (see Table 2 ▸).

### 3.3. Actuator influence   

**Figure 4:**
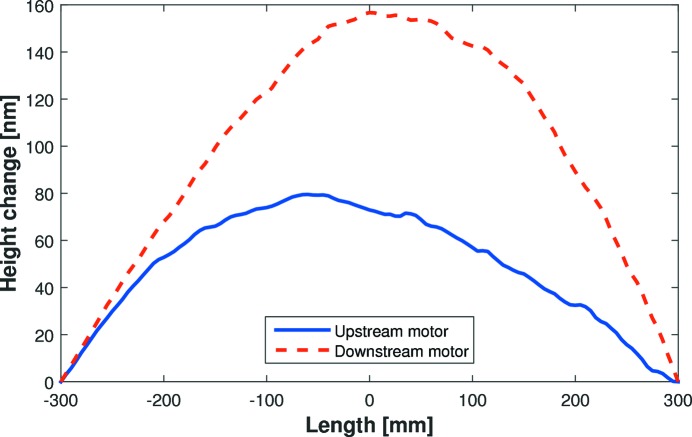
Change in the E-line mirror’s height profile induced by applying +50k counts to the upstream (blue solid curve) or downstream motor (red dashed curve). The asymmetric nature of bending illustrates how elliptical surface profiles and correction of third-order height errors can be achieved by applying unequal forces to the two ends of the mirror.

The major benefit of a two-moment bender is that asymmetric third-order polynomial changes can be made to the mirror’s height profile by applying unequal forces to the ends of the mirror. In addition to achieving elliptical bending, this also enables correction of third-order optical errors from polishing or clamping. To determine the individual influence of each actuator, three scans were performed: the first with equal motor counts applied to both bending motors; and the second and third scans with an additional 50k counts (5% of the central 1000k range) applied only to the upstream or downstream motor, respectively. As shown in Fig. 4 ▸, subtracting the first scan from the second, or the first scan from the third, reveals the individual response of the upstream or downstream motor, respectively. Such curves are comparable with the piezo response functions of deformable bimorph mirrors, illustrating the behaviour of individual piezo actuators to applied voltage. The discrepancy in the two amplitudes is due to the trapezoidal shape of the substrate: the same bending force has a greater effect at the thinner (downstream) end. Assuming that the response functions are linear and independent, the inverse matrix method (Signorato *et al.*, 1998 ▸) enables the correction profile to be decomposed into a linear combination of actuator functions, thereby providing quick predictions about the actuator settings necessary to bend the mirror to a given shape.

**Figure 5:**
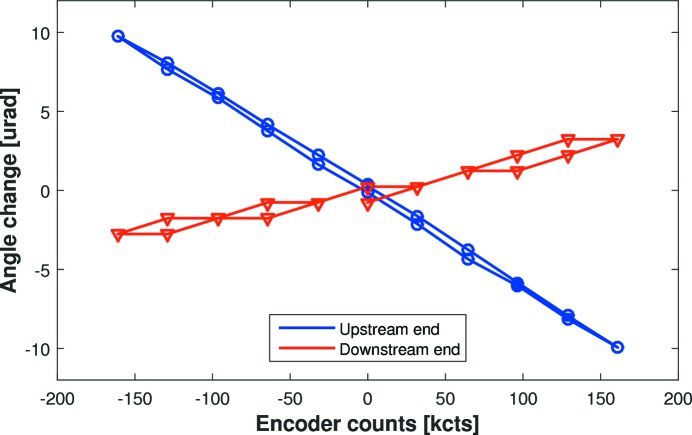
Measurement of local angle changes at the upstream (blue circles) and downstream (red triangles) ends of the C-line mirror as a function of force applied only at the upstream end of the mirror. Parasitic angle changes at the downstream end are about three times smaller than at the upstream end, indicative of coupling.

However, unlike bimorph mirrors, the response functions of the individual mechanical bender motors were found to be intrinsically linked: applying force to one end of the mirror influenced the force applied to the other end. In this instance, perhaps due to non-linearity within the system, or the influence of the spring-loaded compensator, the matrix method of optimization was not successful. In the limited time available, it was decided to concentrate on the simpler optimization method described in §3.4, but this is certainly an area for future study. To investigate how force is physically coupled through the mirror, a series of preliminary experiments were performed at FMB Oxford. An autocollimator (TriAngle UltraSpec TA US 300-57) and angle-measuring interferometer (Renishaw XL-80 using angular optics) measured the local deflection angle of the ends of each mirror in response to force applied only at the upstream end. Fig. 5 ▸ shows that the local angle of the upstream end (blue circles) changed by ∼20 µrad in response to varying the upstream motor over a range of ±170k encoder counts. At the same time, the downstream end (red triangles) of the mirror parasitically changed by 6 µrad, indicating coupling between the two bender motors through the substrate. The angle response was slightly different when bending in the positive and negative directions, indicative of hysteresis.

### 3.4. Elliptical bending and optimization using Diamond-NOM feedback   

**Figure 6:**
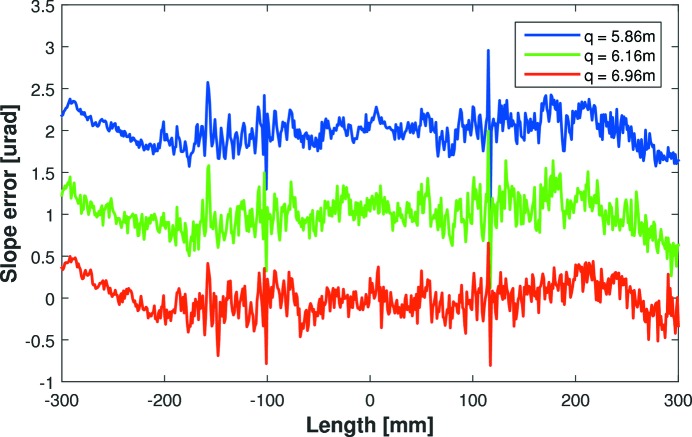
Slope errors curves for the C-line mirror bent to three required ellipses (*q* = 5.86, 6.16 and 6.96 m, all with *p* = 57.9 and θ = 3 mrad), as measured by the Diamond-NOM. Residuals are 185, 194 and 206 nrad r.m.s., respectively (curves offset vertically for clarity). This shows that the clamping and bender mechanisms are not inducing significant distortions to the optical surface.

The simplest method of optimally bending the mirror to a given ellipse is twofold. Firstly, apply equal counts to both bender motors to achieve approximately the correct cylindrical curvature. Since the tangential radius of curvature is approximately proportional to the inverse of the applied bending force, only a few measurements of the radius as a function of motor settings are necessary to quickly calculate the linear proportionality constant. Armed with this knowledge, the mirror can then be bent to the correct curvature. Secondly, to induce ellipticity (asymmetry) in the mirror’s height profile, increase the force applied by one motor and decrease the force applied by the other motor by an equal amount. In practice for elliptical bending, the initial prediction of motor settings typically produces a small height error with incorrect curvature and/or ellipticity. Using metrology feedback, minor changes can be made to the absolute values of both motors, or the difference between them, to iteratively optimize the surface profile to the required ellipse. Using this simple procedure, the C-line mirror was bent and optimized to the three required ellipses (see Table 2 ▸). Fig. 6 ▸ shows that the slope error residuals for the three ellipses were 185, 194 and 206 nrad r.m.s. As anticipated, and as predicted by FEA, the plots (offset vertically for clarity) are very similar. This indicates that the residual errors are dominated by polishing errors on the substrate, and that the bender mechanism is not introducing additional parasitic distortions.

**Figure 7:**
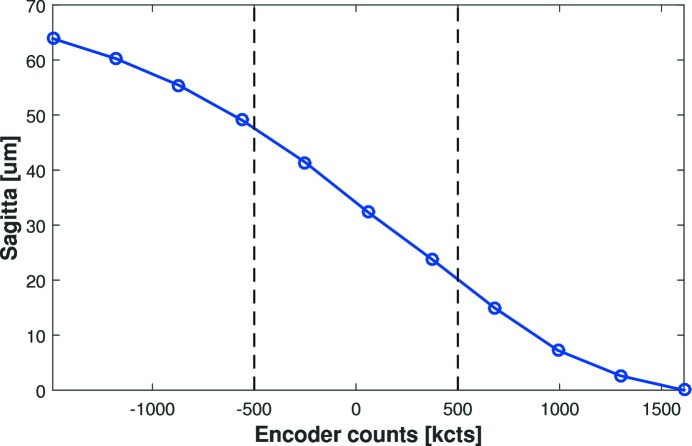
Displacement interferometer measurement of the depth (sagitta) at the centre of the mirror as a function of equal counts applied to both bending motors. Over the central range of ±500k counts, predicted to achieve the required ellipses, the sagitta follows an approximately linear relationship. Hence, to a first approximation, the bending force applied is shown to be proportional to the motor counts.

Without direct measurement of the applied forces (*e.g.* using load cells) it is difficult to know exactly how much force is applied by each motor, and the relative force offset between the two motors. Prior to slope profilometry at Diamond, preliminary tests were performed at FMB Oxford to verify the range and linearity of bending. As shown in Fig. 7 ▸, a displacement interferometer (Renishaw XL-80 with linear optics) quantified how the mirror’s sagitta (depth at the centre of the mirror relative to the two ends) changed as a function of equal counts being applied to both bender motors. For extreme bending, the response is sinusoidal. But over the central bending region (±500k counts) needed to generate the required ellipses, the sagitta and encoder counts follow a linear relationship. To a first approximation, this shows that the applied force is proportional to the motor’s encoder counts.

**Figure 8:**
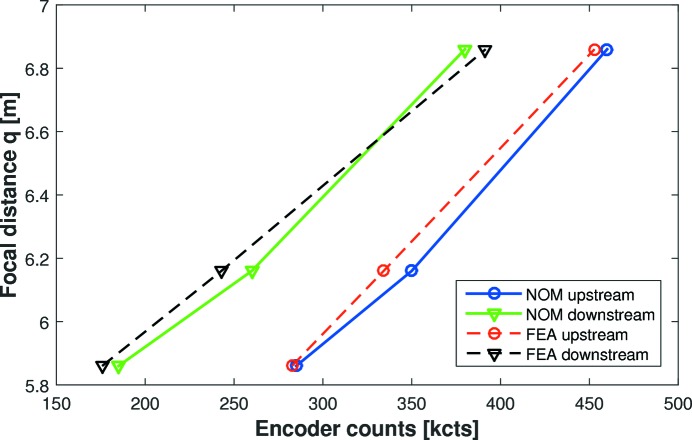
Optimal motor counts for the C-line mirror to achieve the correct focal distance *q* for the three specified ellipses, as measured by the Diamond-NOM (solid lines) and as predicted by FEA (dashed lines). Circles and triangles represent upstream and downstream motor values, respectively. Such relationships enable the beamline user to quickly estimate motor settings which will bend the mirror to any ellipse within the range.

FEA calculations were performed by FMB Oxford to predict the pairs of forces required to optimally bend the C-line mirror to the required ellipses. Based on the assumption that applied force is linear to encoder counts, Fig. 8 ▸ shows the motor settings necessary to achieve a given focal distance *q*, as predicted by FEA and measured by slope profilometry. Such relationships enables the beamline user to quickly estimate motor settings which will bend the mirror to any ellipse within the range.

### 3.5. Bend repeatability and stability   

**Figure 9:**
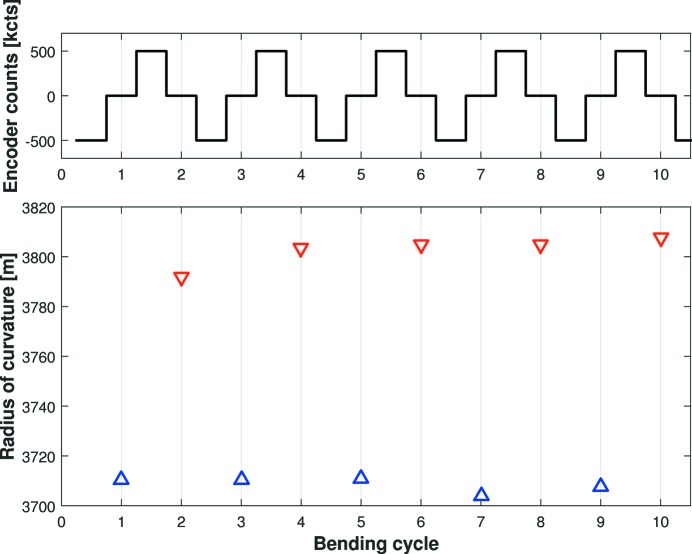
Bending repeatability and hysteresis of the C-line mirror when driven to the nominal ellipse (*q* = 6.16 m) from 500k counts away in either the positive or negative bending direction. The upper chart shows the demanded motor positions relative to the required ellipse, and the lower chart shows the tangential radius of curvature as measured by the Diamond-NOM. Odd- or even-numbered iterations correspond to approaching the required curvature by increasing or decreasing motor counts, respectively. R.m.s. deviation, relative to the average radius, is 0.085% and 0.156% in the two bend directions.

For many beamline experiments, it is vitally important that the X-ray beam size and shape can reliably be achieved and maintained. Fig. 9 ▸ shows the repeatability and hysteresis of the radius of curvature of the C-line mirror when the nominal ellipse (*q* = 6.16 m) is approached from ±500k motor counts in the negative or positive bending directions, after a wait time of ∼20 min (to allow for mechanical settling). As with most mechanical systems, the distinct separation of the two datasets shows that the mirror exhibits bend hysteresis: the mirror bends to a slightly different radius when approached from the two bending directions. However, it can be seen that the mirror reliably returns to the same radius when uni-directionally approached from either the negative [peak-to-valley (PV) = 0.193% of average value, r.m.s. = 0.085%] or positive (PV = 0.384%, r.m.s. = 0.156%) bend directions. Since bending behaviour is very repeatable, such metrology data could be used to actively correct the radius of curvature depending on the direction of bending, which would further improve the repeatability of bi-directional bending.

**Figure 10:**
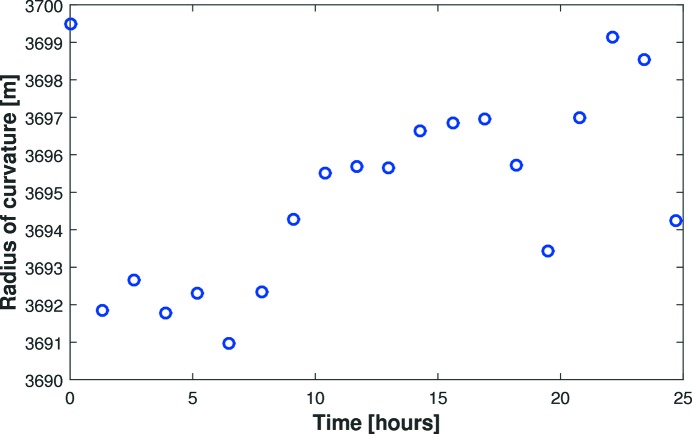
When bent to a given ellipse, the C-line mirror’s radius of curvature drifted by only 0.23% peak-to-valley and 0.07% r.m.s. (relative to the average tangential radius) over a 24 h period.

To investigate stability of bending, the C-line mirror was bent to the nominal ellipse and repeatedly measured over a 24 h period with 20 successive Diamond-NOM scans. Fig. 10 ▸ shows the excellent stability of tangential curvature (relative to the best-fit cylinder) with a peak-to-valley change of 0.23% (relative to the average curvature) and a standard deviation of 0.07%. The 0.2% jump in curvature observed between the first two scans is caused by mechanical settling. Subsequent curvature drifts can likely be attributed to small temperature fluctuations inside the enclosure caused by heat from the motors which were left on throughout the tests. Alternatively, the best-fit ellipse (by optimizing the angle of incidence θ) changed by 0.18%. It is not unreasonable to assume that the mirror’s stability will be further enhanced when operating on the beamline under vacuum.

## 4. Conclusions   

*Ex situ* metrology using the Diamond-NOM slope profilometer has shown that two-moment mechanical actuator systems built by FMB Oxford can successfully bend X-ray mirrors with trapezoidal widths to a range of useful ellipses. The bending mechanism adds minimal deformation to the long (0.9 m) substrates, and state-of-the-art slope error residuals of <200 nrad r.m.s. are achieved over the full elliptical range of bending. Experimental results are in excellent agreement with FEA analysis, and can be used to predict the gravitational sag compensation and the motor counts needed to bend to any ellipse within the working range. The enhanced bending performance compared with a one-moment cylindrical bender could be of great benefit for accurate elliptical focusing of X-rays at many XFEL and synchrotron beamlines. High levels of repeatability and stability of bending are beneficial for beamlines requiring long duration experiments or frequent changes of bending to suit the experimental configuration.

## Figures and Tables

**Table 1:** Parameters for the two optical substrates, as measured facing sideways by Zeiss’ D100 interferometer

Parameter	Mirror 1 (C-line)	Mirror 2 (E-line)
Substrate	Silicon	Silicon
Length *L* (mm)	900	900
Height *H* (mm)	57.5	57.5
Trapezoidal width *W* (mm)	76.3–63.7	77.9–62.1
Active area *L* × *W* (mm)	700 × 50	700 × 20
Tangential slope error r.m.s. (nrad)	170	140
Tangential radius *R* (km)	1010	460

**Table 2:** Elliptical parameters of the two mechanically bent X-ray mirror systems

	Mirror 1 (C-line)	Mirror 2 (E-line)
Source to mirror *p* (m)	57.9	54.38
Mirror to focus *q* (m)	5.86, 6.16, 6.96	4.599, 4.899, 5.699
Angle of incidence θ (mrad)	3	3
